# Towards 3D self-assembled rolled multiwall carbon nanotube structures by spontaneous peel off

**DOI:** 10.3762/bjnano.11.168

**Published:** 2020-12-18

**Authors:** Jonathan Quinson

**Affiliations:** 1 Department of Chemistry, University of Copenhagen, Universitetsparken 5, 2100 Copenhagen Ø, Denmark; 2 Work carried out at: Department of Materials, University of Oxford, Parks Road, Oxford OX1 3PH, United Kingdom

**Keywords:** chemical vapor deposition, multiwall carbon nanotubes, nitrogen doping, peel off, rolled carbon nanotubes

## Abstract

Controlling the 3D assembly of individual nanomaterials can be a challenging task. However, it opens up opportunities for the production of increasingly complex nanostructures. Unusual rolled multiwall carbon nanotube structures are synthesized here by simply inducing a change of precursor composition during the growth of multiwall carbon nanotube forests. The multiwall carbon nanotube structures are comprised of nitrogen-doped and undoped sections, and are obtained via a detailed peel off and roll mechanism. These results open new doors for the development of increasingly complex nanostructures.

## Introduction

Carbon nanotubes (CNTs) are popular materials used in various applications [1]. These tubular hollow carbon nanomaterials have proven to be useful in multiple scientific fields [2–6]. Complex structures with increasingly controlled properties are obtained having CNTs as building blocks. For instance, 3D structures made of CNTs can be synthesized on supports as self-assembled “forests” [7]. These structures have been employed in biomedical applications [7], chromatography [8], or filtration [9]. However, support-free 3D structures typically require extra synthesis steps. “Sponges” or “cages” can be produced by freeze-drying preformed CNTs [10] or by etching template materials [11].

A way to tune CNT properties further is to introduce other elements in the carbon network (e.g., nitrogen [12–14]). This so-called doping tunes CNT properties for specific applications. A step further to design more complex CNTs is to develop materials with a composition change (e.g., CNT structures displaying both properties of nitrogen-doped and undoped carbon along the same CNT [15]). A structural change in CNTs is achieved by tuning experimental parameters during CNT synthesis [16].

To date, CNTs with so-called intratubular junctions have been used exclusively for electronic applications due to their p–n junction behavior [17–19]. The potential of CNTs with junctions to serve as building blocks for other more complex nanomaterials remains underexploited. We recently proposed new approaches to prepare such complex materials [16]. In this work it is explored how template-free 3D nanostructures can be obtained by using CNTs with junctions.

## Results

To develop new ways to induce a compositional change within CNTs and to optimize the formation of complex CNT structures, the possibility to couple aerosol-assisted chemical vapor deposition (AACVD) [20] and chemical vapor deposition (CVD) was explored [16]. For detailed synthesis and characterization protocols, including control experiments, please see Experimental section. Undoped sections of multiwall CNTs (MWCNTs) grown from toluene or acetylene, are referred as “C” whereas nitrogen-doped sections, grown under ammonia-containing atmosphere or using benzylamine, are referred as “N” [21]. The chronological order of the grown sections is shown by the index with an ascending numerical order. Therefore, the two synthesis sequences described here lead to C_1_/N_2_/C_3_ and C_1_/N_2_/C_3_/N_4_ structures.

The coupling of AACVD and CVD leads to MWCNTs with a compositional change [16]. In addition to obtaining the expected vertically aligned MWCNT forests or carpets [22], less conventional and complex structures were also developed and their formation mechanism is proposed in this study. In a typical AACVD synthesis, MWCNTs grow perpendicular to the substrates (i.e., quartz reactor walls or silicon wafers [23]). Under the conditions of the present study, it is observed, by the naked eye, that layers peel off from the MWCNT forests after synthesis. This is observed without the need to apply any mechanical stress. However, when this phenomenon occurs, it is not possible to identify an area without MWCNTs. In other words, despite the peeling off the substrates remain dark, which indicates that MWCNTs are still present.

**Figure 1:**
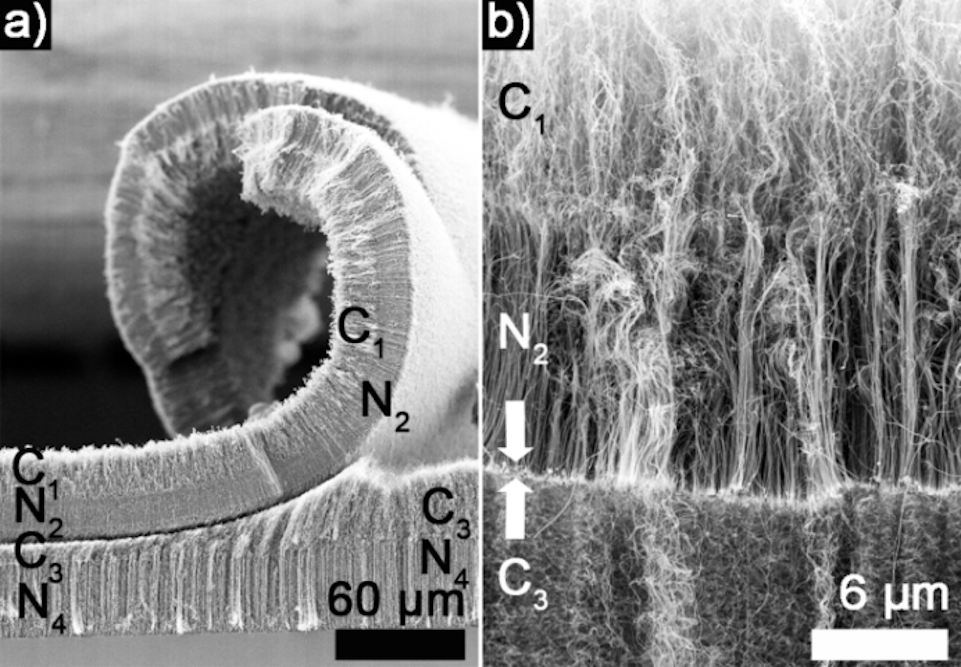
(a) SEM micrograph of a C_1_/N_2_/C_3_/N_4_ structure in which a break between the N_2_ and C_3_ part leads to MWCNTs layers to peel off. (b) SEM micrograph of C_1_/N_2_/C_3_ structures in which the N_2_/C_3_ interface displays iron-based particles. The bright line between the two arrows in the image is associated with a strong morphological change from straight to wavy MWCNTs.

In order to characterize the nature of the peeled-off layer, scanning electron microscopy (SEM) characterization was performed. Figure 1 displays an SEM micrograph of a sample with a C_1_/N_2_/C_3_/N_4_ structure in which the peeling off was first observed by the naked eye. Based on a morphological characterization from a previous work [16], four distinct sections are identified along the MWCNT forest. From top to bottom (bottom is marked by the silicon substrate at the bottom of the image), one can see a “wavy”, a “straight”, another “wavy”, and a final “straight” section. A wavy structure is the expected morphology for undoped MWCNTs, whereas a straight section is expected for N-doped MWCNTs [6,16,21]. Thus, the sequence observed is in agreement with the synthesis sequence performed and with a root-growth mechanism previously detailed [16,24–25]. It is also in agreement with the Raman characterization detailed below.

The SEM analysis reveals that the peeled-off layers are C_1_/N_2_ sections detached from another supporting C_3_/N_4_ MWCNT layer. This implies that only a few layers of MWCNTs are disconnected from the forest. Such peeling off is not observed when no compositional change is induced (i.e., if only undoped or nitrogen-doped MWCNTs are synthesized [21]). The peeling off seems to happen preferentially at a N*_x_*/C*_x_*_+1_ interface, which is weaker and less continuous than a C*_x_*/N*_x_*_+1_ interface, as we recently documented [16] and as further detailed in the Discussion section.

According to the hypothesis that the peeling off is promoted by a challenging formation of N*_x_*/C*_x_*_+1_ junctions, the C_1_/N_2_/C_3_ structure was studied further. The SEM micrograph of a C_1_/N_2_/C_3_ structure (Figure 1b) shows three different sections: From top to bottom, there is a wavy, a straight, and another wavy section. This sequential changes in the morphology is in agreement with the synthesis sequence used. The interface between the expected N_2_ and C_3_ sections is marked by a brighter line.

**Figure 2:**
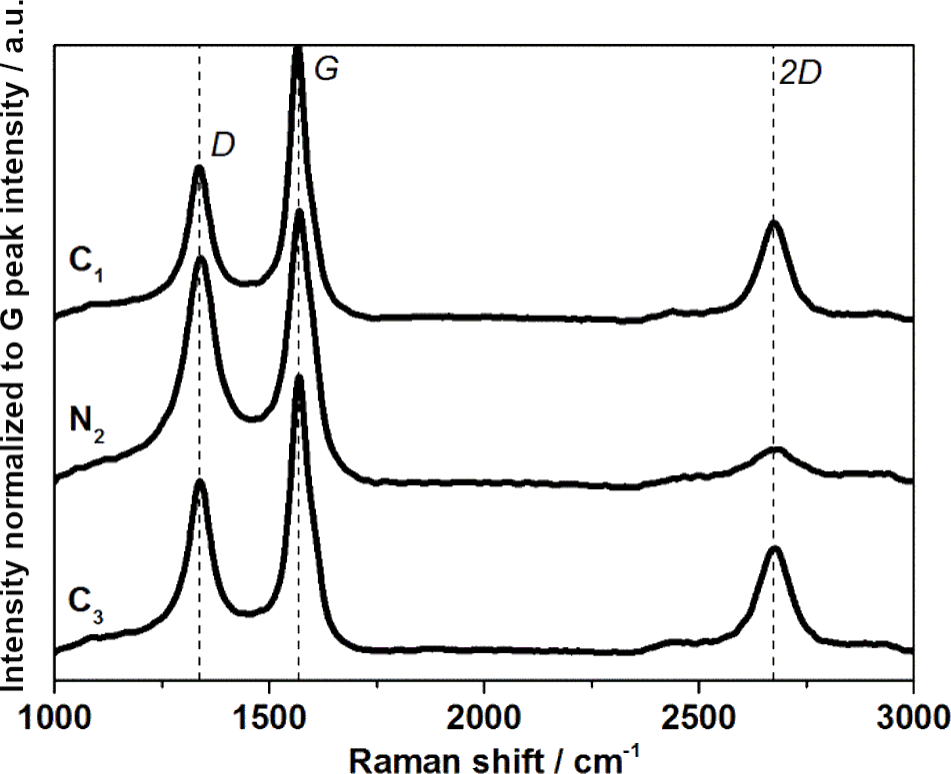
Raman spectra of each section of a C_1_/N_2_/C_3_ structure.

Raman spectra of undoped MWCNTs and nitrogen-doped MWCNTs are significantly different [6,16,21]. Undoped MWCNTs are characterized by an intensity ratio between D and G peaks smaller than 1 and by a strong 2D peak, whereas nitrogen-doped MWCNTs show an intensity ratio between D and G peaks of approx. 1 and a less pronounced 2D peak [12,21]. The D, G and 2D peaks of a C_1_/N_2_/C_3_ structure is shown in Figure 2. The D peak refers to a defect in the MWCNT structure. An intense D peak (relative to the G peak intensity) correlates to higher defects, for instance, induced by nitrogen doping. The G and 2D peaks are related to the graphitization of MWCNTs. An intense G peak (relative to the D peak intensity) and the presence of a 2D peak indicate a more graphitized MWCNT.

Conveniently, the structures obtained here have sections that are long enough (>10 μm) to be unambiguously focused by a Raman laser, using the optical lens of a Raman microscope [16]. Typical Raman spectra features obtained along C_1_/N_2_/C_3_ structures are shown in Figure 2. In good agreement with the synthesis sequence performed, the Raman spectra show typical features of undoped MWCNTs for the expected C sections, and typical features of nitrogen-doped MWCNTs for the expected N sections. These characteristic features in the Raman spectra were observed in all MWCNT structures with different sections investigated in this study.

**Figure 3:**
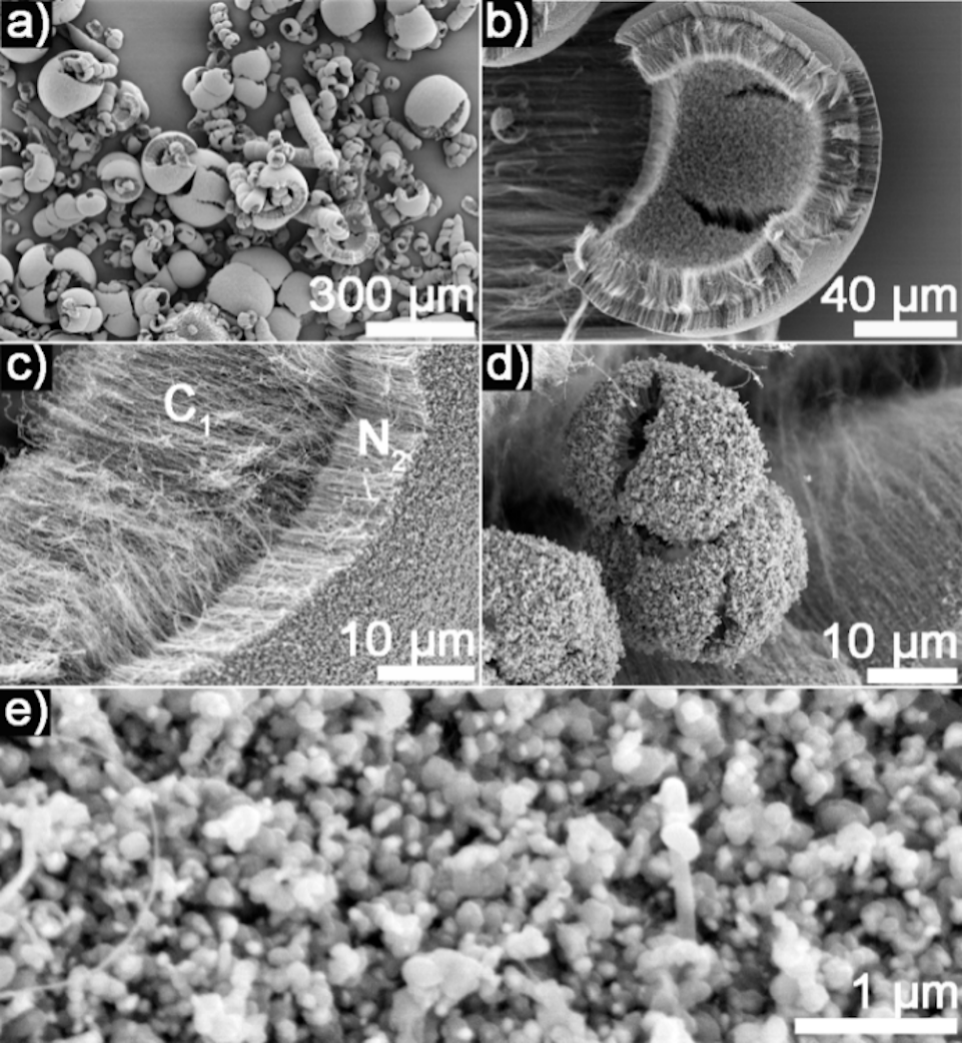
(a–e) SEM micrographs of the powder collected after the synthesis of C_1_/N_2_/C_3_ structures. (e) The outer surface of the rolled structure, brighter in the SEM micrograph, is made of iron catalyst particles.

Under the experimental conditions leading to the C_1_/N_2_/C_3_ structure, more peeling off is observed. After synthesis, a simple bending of the quartz tube, which is used as a reactor, enables the collection of several milligrams of a black powder. SEM micrographs of the collected powder are shown in Figure 3. The powder observed is not mechanically detached from any substrate where the MWCNT growth is expected to occur. Conversely, this material is likely to be MWCNTs that peeled off from the carpet and detached from MWCNT underlayers, with the latest layer still connected to the substrate (Figure 1a).

## Discussion

**Figure 4:**
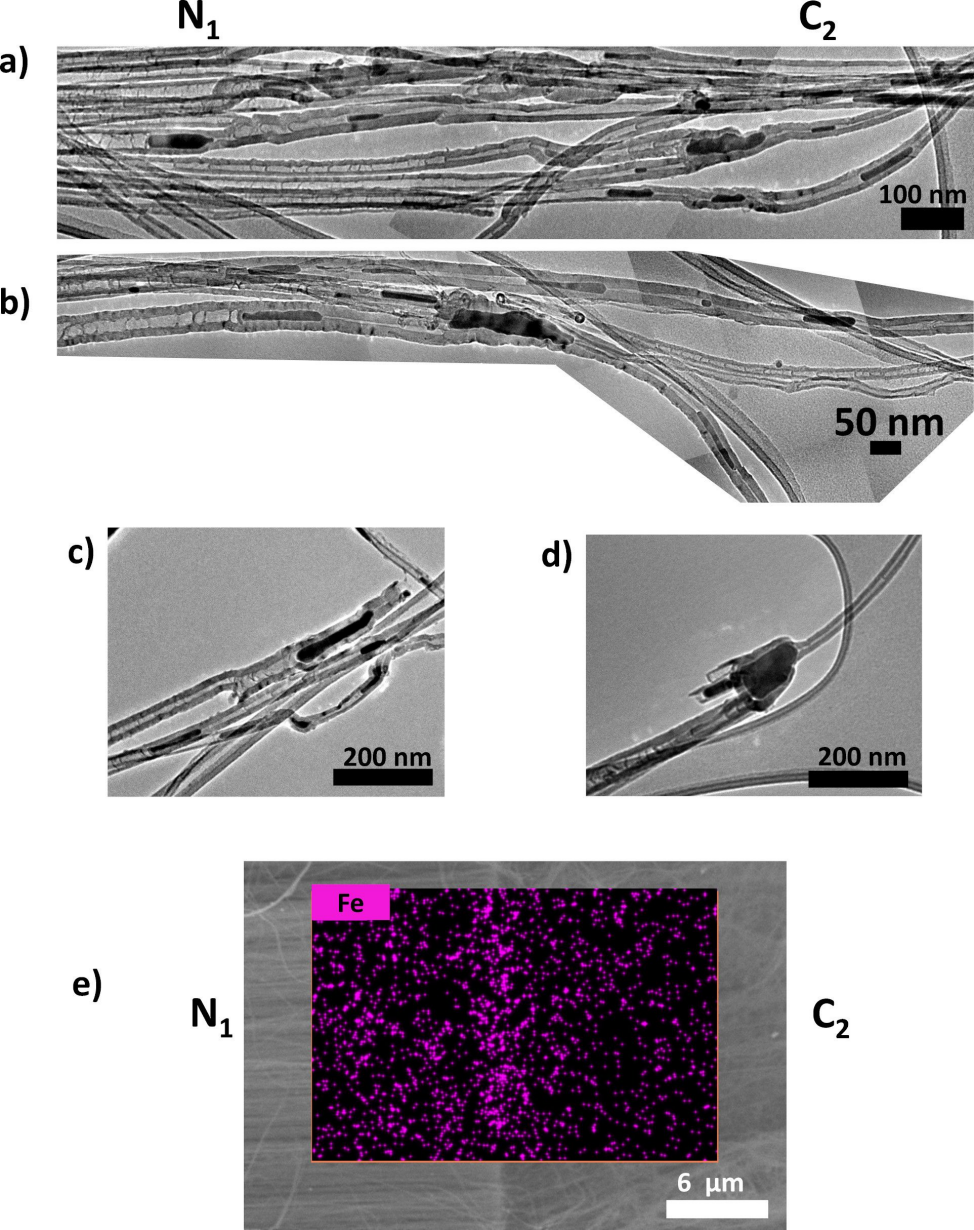
(a–d) TEM characterization of a N_1_/C_2_ interface marked by the presence of metal (iron) nanoparticles before and after a clear change in the typical structure of N (larger inner diameter and corrugated structure) and C (smaller inner diameter) sections. Panels (a,d) were obtained by superimposing several TEM micrographs, all taken at the same magnification along the N_1_/C_2_ interface. (c,d) Examples of individual MWCNTs with a clear change of structure before and after the interface, which is marked by a nanoparticle (dark structure in the TEM images). (c) Broken MWCNT structure after the interface, whereas in (d) the structure remains intact. (e) SEM backscattered images and the related Fe EDS mapping at a N_1_/C_2_ interface.

To clarify the formation mechanism of the unusual structures shown in Figure 3, control experiments were conducted in which only C_1_/N_2_ or C_2_/N_1_ structures were formed. SEM characterization of the related materials is reported in Supporting Information File 1, Figure S1 and Figure S2. In these control experiments, no rolled structures similar to those reported in Figure 3 were observed. The C_1_/N_2_ structures are more continuous than the C_2_/N_1_ structures. At the root of the C_1_/N_2_ forest, structures compatible with relatively large nanoparticles (approx. 100 nm) are identified. These are attributed to Fe metal catalysts formed during the synthesis and from which MWCNTs grow, assuming a root-growth mechanism [16,26]. For N_1_/C_2_ structures, it is not straightforward to observe the same structures at the bottom of the CNT forest, although clear structures related to Fe nanoparticles are observed at the N_1_/C_2_ interface (see discussion below Figure 4). This confirms that creating a N_1_/C_2_ interface is challenging.

It is established that the inner diameter of MWCNTs increases with the diameter of the nanoparticle from which they grow [26]. It is established that N-MWCNTs have a larger inner diameter than MWCNTs without an intended doping [6,12,21]. This is also observed in N_1_/C_2_ structures (Figure 4) in which the N section has a larger inner diameter and the C section has a smaller inner diameter [16]. Thus, the size of a catalyst to grow a N-MWCNT is larger than that to grow an undoped C-MWCNT. This size is determined by the experimental conditions, such as the presence or absence of N atoms. This is related to the fact that smaller nanoparticles are more difficult to be identified at the root of MWCNTs in N_1_/C_2_ junctions than larger nanoparticles at the root of MWCNT in C_1_/N_2_ junctions (Supporting Information File 1, Figure S1).

**Figure 5:**
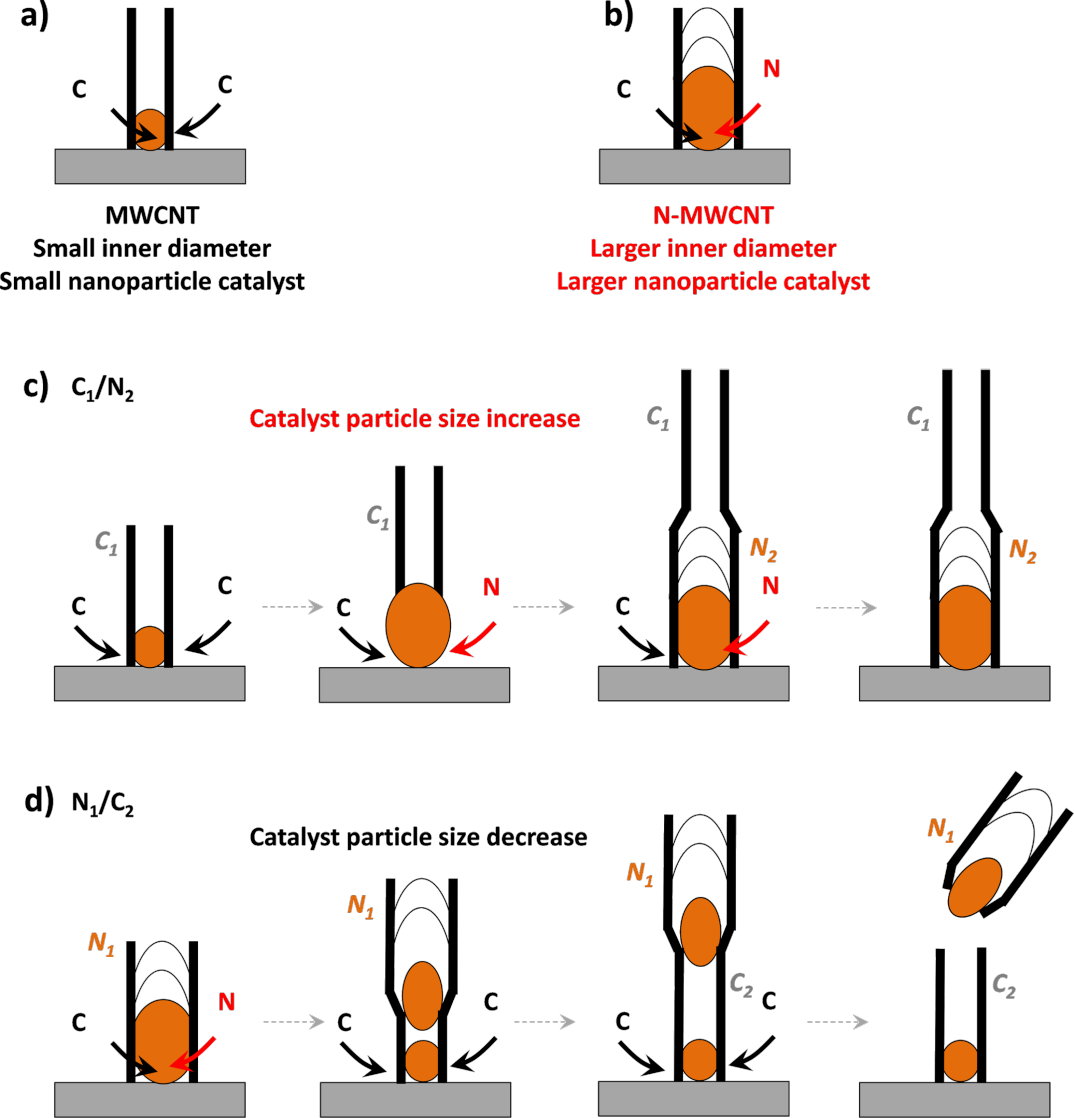
Schematic representing the growth mechanism and the relation between inner diameter and catalyst nanoparticle size and position for (a) MWCNT, (b) N-MWCNT, (c) C_1_/N_2_, and (d) N_1_/C_2_ MWCNT structures.

A C_1_/N_2_ junction, therefore, requires a size increase of the nanocatalyst whereas an N_1_/C_2_ junction requires a size decrease of the nanocatalyst. For the synthesis performed, a size increase is relatively easy to be achieved since ferrocene, source of the iron atoms, is constantly provided (see Experimental section). However, a size decrease is possible mainly by a size change of the catalyst nanoparticle and by some “loss” of the catalyst (e.g., within the MWCNT). This scenario is supported by EDS and TEM data (Figure 4). Nanoparticles with higher atomic weight ratio appear as white features in SEM micrographs and as dark features in TEM micrographs. SEM characterization of vertically aligned MWCNTs, together with Raman spectra acquired on the same cross section, unambiguously assign a given morphology to a given structure in a confirmed root-growth mechanism, since the sample can be kept on a substrate [16]. TEM characterization is more challenging since the information regarding the relative position of the different sections is lost during sample preparation. Nevertheless, on MWCNT structures with only one junction a change in structure can still be related to the precursor used. In the case of N_1_/C_2_ junctions (Figure 4a and Figure 4b) the interface between the two expected sections is conveniently marked by darker features, which correspond well to the lighter features observed in SEM micrographs (Supporting Information File 1, Figure S1 and Figure S2). These features are related to iron-based nanoparticles, as confirmed by EDS. An area with higher intensity of Fe is observed at the interface of a N_1_/C_2_ structure (Figure 5e) as reported in our previous work [16].

On each side of the nanoparticle marking the N_1_/C_2_ interface, the inner diameter of the tube is drastically changed. One side shows a relatively large inner diameter (in agreement with the N-MWCNT structure) whereas the other side shows a relatively small inner diameter (in agreement with undoped MWCNT). It is also noted that on the side of the smaller inner diameter, the MWCNT structure is often broken. Figure 4c highlights the weaker nature of the N_1_/C_2_ interface.

Taking all this information into account, a mechanism for the formation of C_1_/N_2_ and N_1_/C_2_ structures is suggested (Figure 5). This schematic representation is based on the experimental results of a confirmed root-growth mechanism, on the relationship between the size of the catalyst particle, and on the diameter of MWCNTs and N-MWCNTs. In addition, it is in agreement with [6,12,16,20–21,24,26–30] and it builds up upon the formation of C_1_/N_2_ and N_1_/C_2_ structures, as proposed in [16]. The need for a size decrease to form a N_1_/C_2_ junction acts as a constraint on the MWCNT structure, leading to a weaker junction that often breaks (which is not easily observed in a C_1_/N_2_ junction).

In the case of only one junction, no peeling off is observed (Supporting Information File 1, Figure S1 and Figure S2, and [16]). Considering that MWCNT forests of vertically aligned N-MWCNTs or C-MWCNTs are easily obtained [12,21], it is expected that, even in the case of a weak junction that ultimately breaks, the two sections will simply stay on top of each other. In order to promote the roll-up, the section to be developed cannot be uniform and the layer that is peeled off needs to show a more complex structure. In this work, C_1_/N_2_ junctions with different lengths are examples of suitable complex structures. It is clear from the SEM characterization that the structure of N-MWCNTs is different from the structure of C-MWCNTs: It is curly for C sections and straight for N sections (Figure 1, Supporting Information File 1, Figure S1 and Figure S2, and [16]). This is likely related to different interactions between different sections. When a composition change is performed and a N*_x_*/C*_x_*_+1_ is added to a C*_x−_*_1_/N*_x_* structure, the detachment of the N*_x_* section from the C*_x_*_+1_ section is favored (Figure 1 and Figure 5). Since C*_x_*_−1_/N*_x_* is stronger and more continuous, its entire structure peels off and, thus, becomes free-standing. Different interactions between C*_x_*_−1_ and N*_x_* sections [31] lead to the rolling of the free-standing structure, which eventually leads to the MWCNT rolled forest configuration.

Another observation that strongly supports the proposed formation mechanism is the curved and rolled aspect of the structures (Figure 3a–d) and the fact that the outer part of the rolled MWCNTs is made of particle-like structures (Figure 3e). These iron nanoparticles formed as catalyst from ferrocene, under certain reaction conditions [21], appear in the SEM micrographs as brighter features and are typically found at the N*_x_*/C*_x_*_+1_ interface (Figure 1b, Figure 4, Figure 5, Supporting Information File 1, Figure S1 and Figure S2, and [16]). In Figure 1b, these features can be observed at the N_2_/C_3_ interface. After peeling off and rolling (Figure 1a), the N section of the broken N*_x_*/C*_x_*_+1_ interfaces becomes the outer surface of the observed rolls. Therefore, it is not surprising that the presence of iron particles at the N*_x_*/C*_x_*_+1_ interface leads to the presence of iron particles on the outer surface of the obtained rolled C*_x_*_−1_/N*_x_* structures.

In summary, the rolled structures reported here display a curvature that is not usually reported for MWCNT forests or MWCNTs grown under a hydrocarbon flow [21,23]. Control experiments show that creating C_1_/N_2_ or N_1_/C_2_ interfaces does not lead to rolled structures (Supporting Information File 1, Figure S1 and Figure S2). Thus, two junctions are necessary to develop complex structures. The formation of MWCNT structures with a spherical shape is likely derived from a combination of peeling off and rolling phenomena. The peeling off is achieved by developing weak interfaces between different sections of a MWCNT forest. Developing a N*_x_*/C*_x_*_+1_ is an example of how to develop such a weak interface. The driving force behind the rolling phenomena is not yet fully established. However, it is strongly related to the formation of a C*_x_*_−1_/N*_x_* interface in which the length of the C and N sections may play a role. More importantly, different interactions are likely to be developed between MWCNTs within the same section, as indicated by the different morphologies, different diameters, and the apparent packing of MWCNTs. This eventually promotes the resulting spherical geometry by inducing different mechanical forces between the MWCNTs as they become free-standing after the peel off.

## Conclusion

In this study, the controlled synthesis and characterization of rolled self-assembled structures made of MWCNTs is presented. The formation of these structures relies on the formation of MWCNT forests with different sections showing different compositions along the forest, and on the development of a weaker interface between some sections, such as a change in composition from nitrogen-doped to undoped MWCNTs. A suggested peel off and roll formation mechanism is detailed. The results presented here open routes for the production of complex support-free and template-free 3D structures made of MWCNTs with intratubular junctions between sections of different compositions. The approach introduced paves the way for a simple design of new complex 3D sphere-like MWCNT nanostructures whose properties are yet to be fully explored.

## Experimental

### Synthesis

MWCNTs with junctions were prepared by following a general approach detailed recently [16]. MWCNTs were synthesized at 800 °C with argon (Ar, 99.999%, BOC) as the carrier flow using an AACVD setup [12,23]. Briefly, MWCNTs were grown on silicon wafer substrates (10 × 20 mm^2^, Sibert, UK) by using an aerosol CVD setup consisting of a piezoelectric generator, a quartz tube (2.2 cm inner diameter), a 50 cm long horizontal tube furnace, a gas flow controller, and an acetone gas trap. The total gas flow was 2500 sccm for all experiments. In order to obtain different compositions within the MWCNTs, different sequences of different precursors were used during the same synthesis protocol. The length of the different sections was controlled by changing the time of injection of the relevant precursors (the longer the time of injection, the longer the section).

In a first approach, the synthesis of C_1_/N_2_/C_3_ structures was performed as follows. First, a liquid precursor consisting of toluene (99.9%, Sigma-Aldrich) and 5 wt % of ferrocene (98%, Aldrich, purified via sublimation at 90 °C prior to use) was injected into the furnace with Ar gas flow at 2000 sccm in a first precursor line (Supporting Information File 1, Figure S3) for 3 min. In a second precursor line, 500 sccm of Ar were used. Next, while the same ferrocene and toluene mixture was injected into the furnace, ammonia (≥99.98%, BOC) was added to the carrier gas at 30 sccm for 15 min. Finally, acetylene (industrial grade, BOC) was used as a gas precursor at 100 sccm with hydrogen (≥99.995%, BOC) at 30 sccm in an argon flow for 20 min. For this last step, the injection of toluene and ferrocene mixture was stopped. The catalyst for the formation of MWCNTs was formed from the initially introduced ferrocene precursor [21].

In a second approach, the synthesis to obtain C_1_/N_2_/C_3_/N_4_ structures was as follows (Supporting Information File 1, Figure S4). First, a liquid precursor consisting of toluene and 5 wt % of ferrocene was injected into the furnace for 5 min. Next, a solution of benzyalmine (≥99%, Fluka) without ferrocene was injected for 15 min. Then, the same solution of toluene and 5 wt % of ferrocene was used as a precursor and injected for 10 min. Finally, a solution of benzyalmine without ferrocene was injected for 20 min.

In control experiments only two steps were used. As a first step of one type of control experiment, 5 wt % of ferrocene in toluene was continuously injected, for 7 min, in the hot part of the furnace using an Ar flow of 2000 sscm in the first precursor line, while 500 sccm of Ar was mixed with this flow at the furnace entrance of the second precursor line (Supporting Information File 1, Figure S3, step 1). In the second step the conditions in the first line were unchanged whereas the second line allowed a flow of NH_3_ at 30 sccm with 470 sccm of Ar for 30 min (Supporting Information File 1, Figure S3, step 2). In another type of control experiment, the two previous steps were performed in the opposite order. In the first step, 5 wt % of ferrocene in toluene was injected with 2000 sccm Ar in the first line while 470 sccm Ar and 30 sccm NH_3_ were flown in the second line for 15 min. In the second step, 5 wt % of ferrocene in toluene was injected with 2000 sccm Ar in the first line while 500 sccm Ar was flown in the second line for 4 min.

### Characterization

MWCNT structures were characterized by SEM (Jeol 840F operated at 5 kV and a Zeiss NVision FIB microscope equipped with an in-lens and a backscattered-electron detector, also operated at 5 kV) and by Raman spectroscopy (JY Horiba Labram Aramis imaging confocal Raman microscope equipped with a 532 nm green laser). The Raman spectra recorded were consistent within each section with the characteristic spectra of undoped or N-doped MWCNTs. The materials were collected from the furnace after synthesis and deposited onto suitable holders in powder or in as-prepared form on the silicon wafers. For clarity, cross section images of MWCNT forests were obtained by simply cleaving the silicon wafer after MWCNT growth, in order to expose the inside of the MWCNT forests for both Raman and SEM. This was simply achieved by applying mechanical pressure on the edge of the silicon wafer. EDS mapping and associated SEM backscattered images were recorded with a Hitachi TM3000 table top SEM operated at 15 kV. For Fe mapping, a 6.399 keV peak intensity was used. Further characterization details can be found in [16]. Nitrogen doping is expected to be in the range of 0.5–2 atom %, according to previous reports [12,21,32].

## Supporting Information

File 1 Experimental section.
Schemes of the synthesis approaches and further SEM characterization.
