# Supplementary material for: Towards 3D self-assembled rolled multiwall carbon nanotube structures by spontaneous peel off
Source: Beilstein J Nanotechnol. 2020 Dec 18;11:1865–72. doi: 10.3762/bjnano.11.168 (PMC7753106; doi:10.3762/bjnano.11.168)
Supplement: File 1 — Experimental section. Schemes of the synthesis approaches and further SEM characterization. [file Beilstein_J_Nanotechnol-11-1865-s001.pdf]

BEILSTEIN JOURNAL OF NANOTECHNOLOGY

# Supporting Information

for

# Towards 3D self-assembled rolled multiwall carbon nanotube structures by spontaneous peel off

Jonathan Quinson

*Beilstein J. Nanotechnol.* **2020,** *11,* 1865–1872. doi:10.3762/bjnano.11.168

## Experimental section.

## Schemes of the synthesis approaches and further SEM characterization

License and Terms: This is a supporting information file under the terms of the Creative Commons Attribution License (https://creativecommons.org/licenses/by/4.0). Please note that the reuse, redistribution and reproduction in particular requires that the author(s) and source are credited and that individual graphics may be subject to special legal provisions.
The license is subject to the *Beilstein Journal of Nanotechnology* terms and conditions: (https://www.beilstein-journals.org/bjnano/terms)

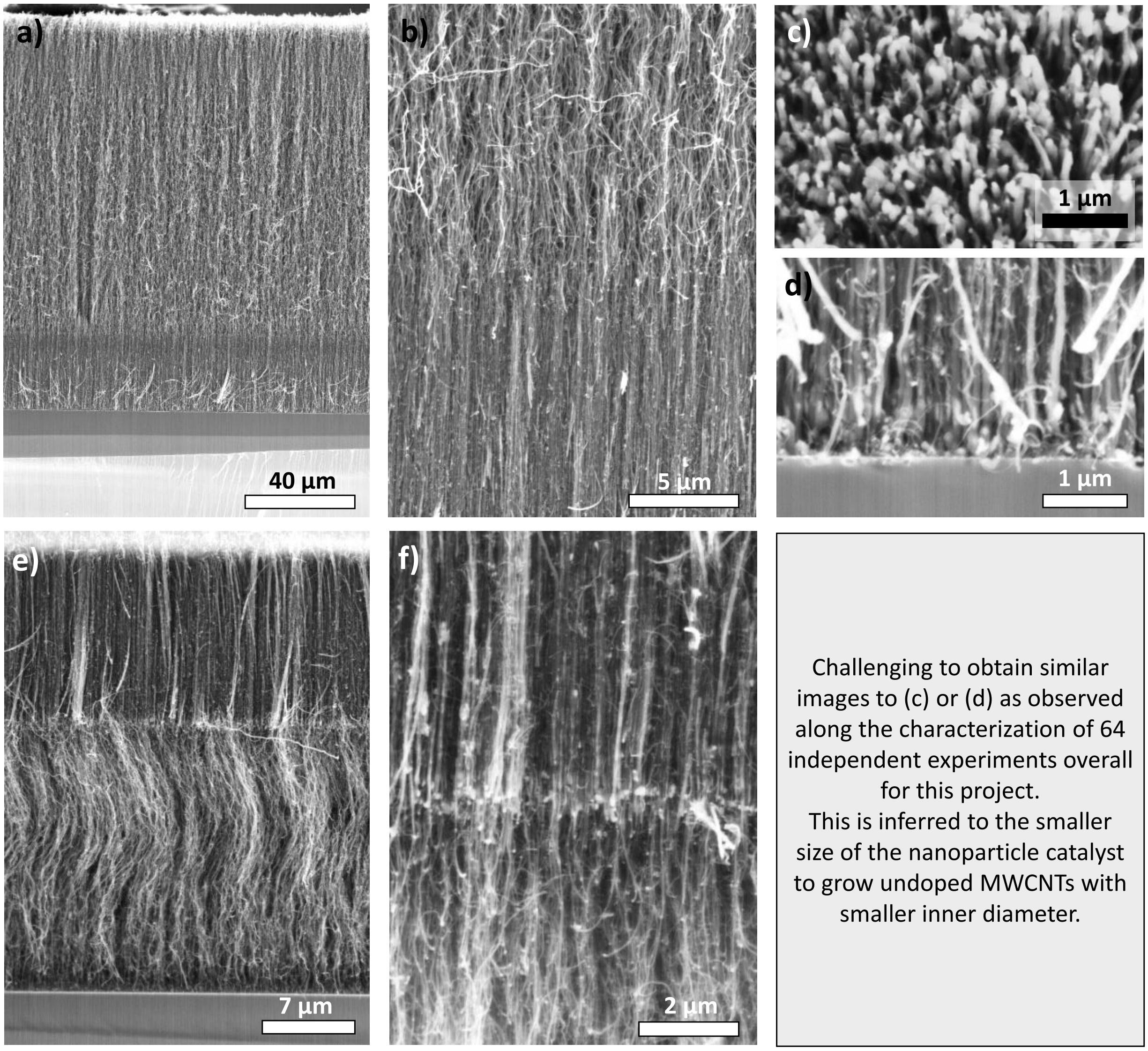


**Figure S1:** SEM characterization of (a–d) MWCNTs with $C_1/N_2$ junctions. The substrate from which the structure grows is at the bottom of the image. (c) Represents the bottom of the MWCNT forest initially in contact with the substrate whereas (d) represents the same part of the MWCNTs still connected to the substrate. (e, f) SEM characterization for MWCNTs with $N_1/C_2$ junctions. All structures were obtained by a liquid–[liquid+gas] approach.

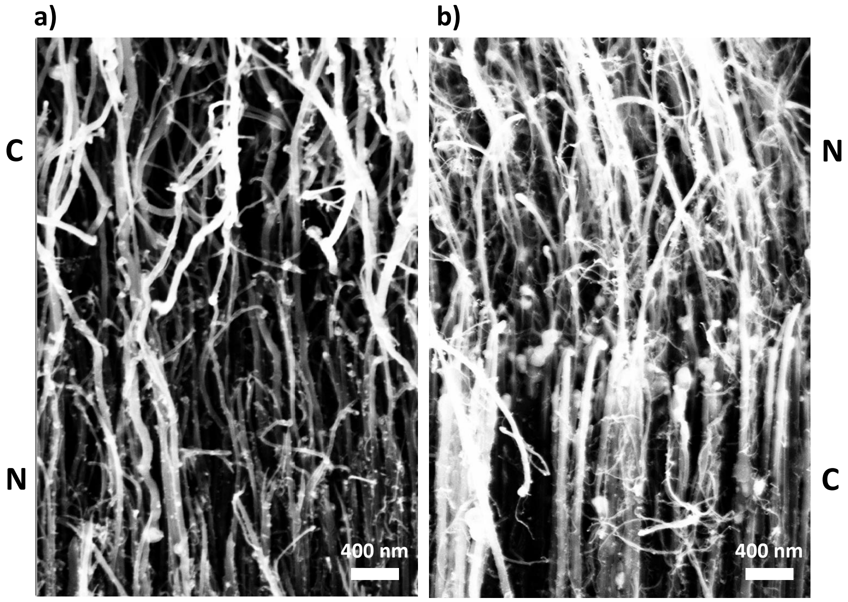


**Figure S2:** SEM characterization of MWCNT with (a) $C_1/N_2$ and (c) $N_1/C_2$ junctions. The orientation and samples are the same as for Figure S1.

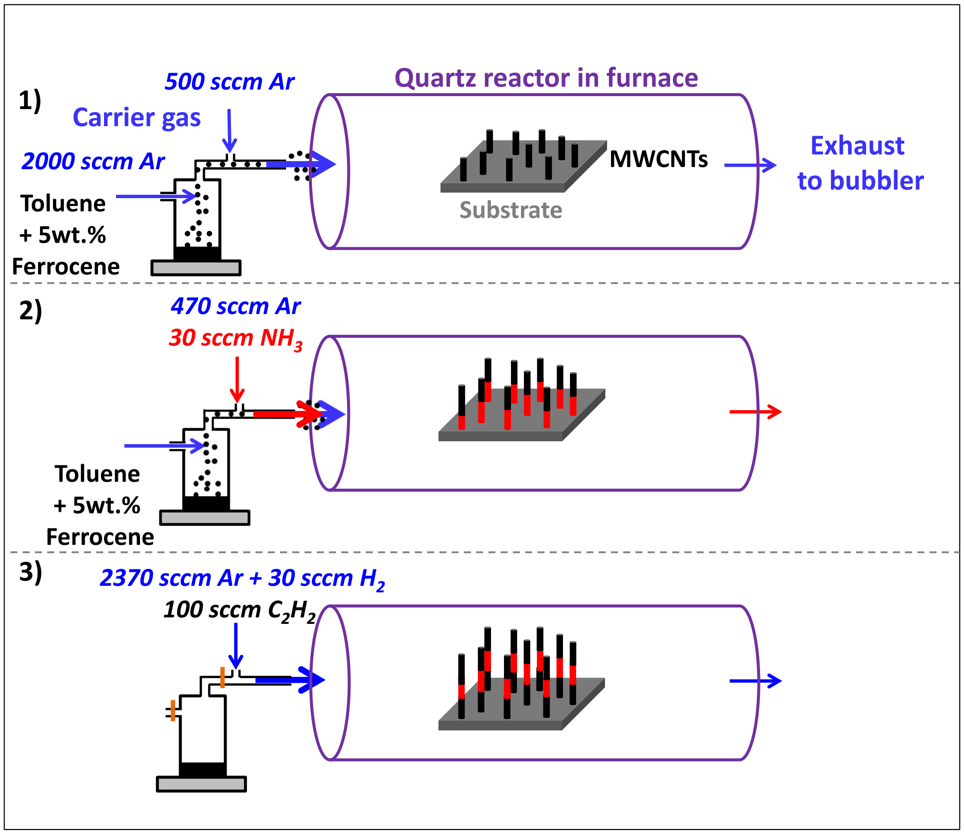


**Figure S3:** Schematic illustration of the liquid–[liquid+gas]–gas synthesis approach. Adapted with permission from Quinson et al. *ACS Applied Nano Materials* **2020**, *3*, 7554–7562. Copyright 2020 American Chemical Society.

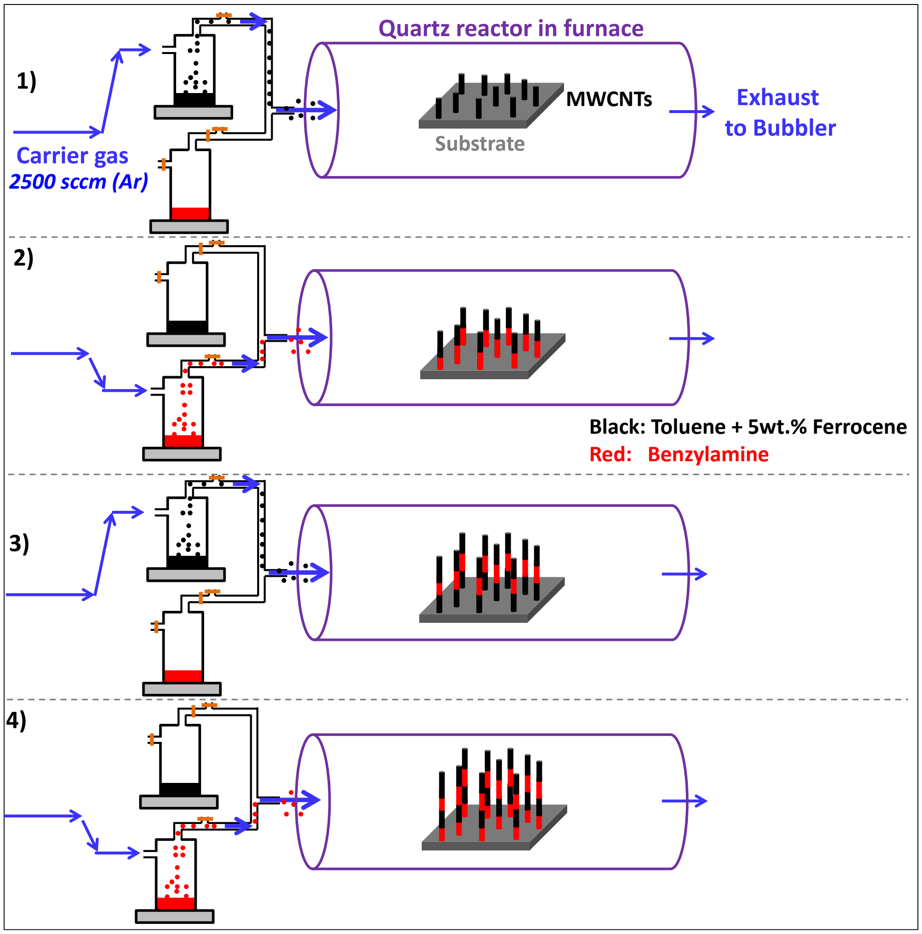


**Figure S4:** Schematic illustration of the liquid–liquid–liquid–liquid synthesis approach. Adapted with permission from Quinson *et al. ACS Applied Nano Materials* **2020**, *3*, 7554–7562. Copyright 2020 American Chemical Society.
